# Burden of hospitalized childhood community-acquired pneumonia: A retrospective cross-sectional study in Vietnam, Malaysia, Indonesia and the Republic of Korea

**DOI:** 10.1080/21645515.2017.1375073

**Published:** 2017-11-10

**Authors:** Kah Kee Tan, Duc Anh Dang, Ki Hwan Kim, Cissy Kartasasmita, Hwang Min Kim, Xu-Hao Zhang, Fakrudeen Shafi, Ta-Wen Yu, Emilio Ledesma, Nadia Meyer

**Affiliations:** a Department of Pediatrics, Tuanku Ja'afar Hospital, Seremban, Negeri Sembilan, Malaysia; b Department of Bacteriology, National Institute of Hygiene and Epidemiology, Hanoi, Vietnam; c Department of Pediatrics, Yonsei University College of Medicine, Severance Children's Hospital, Seoul, Republic of Korea; d Department of Child Health, Faculty of Medicine, University Padjadjaran, Bandung, Indonesia; e Department of Pediatrics, Yonsei University Wonju College of Medicine, Wonju, Republic of Korea; f GSK, Bangalore, Karnataka, India; g GSK, Singapore, Singapore; h GSK, Wavre, Belgium

**Keywords:** Asia, children, costs, epidemiology, hospitalization, pneumonia

## Abstract

**Background**: Few studies describe the community-acquired pneumonia (CAP) burden in children in Asia. We estimated the proportion of all CAP hospitalizations in children from nine hospitals across the Republic of Korea (high-income), Indonesia, Malaysia (middle-income), and Vietnam (low/middle-income).

**Methods**: Over a one or two-year period, children <5 years hospitalized with CAP were identified using ICD-10 discharge codes. Cases were matched to standardized definitions of suspected (S-CAP), confirmed (C-CAP), or bacterial CAP (B-CAP) used in a pneumococcal conjugate vaccine efficacy study (COMPAS). Median total direct medical costs of CAP-related hospitalizations were calculated.

**Results:** ***Vietnam*** (three centers): 7591 CAP episodes were identified with 4.3% (95% confidence interval 4.2;4.4) S-CAP, 3.3% (3.2;3.4) C-CAP and 1.4% (1.3;1.4) B-CAP episodes of all-cause hospitalization in children aged <5 years. The B-CAP case fatality rate (CFR) was 1.3%. ***Malaysia*** (two centers): 1027 CAP episodes were identified with 2.7% (2.6;2.9); 2.6% (2.4;2.8); 0.04% (0.04;0.1) due to S-CAP, C-CAP, and B-CAP, respectively. One child with B-CAP died. ***Indonesia*** (one center): 960 CAP episodes identified with 18.0% (17.0;19.1); 16.8% (15.8;17.9); 0.3% (0.2;0.4) due to S-CAP, C-CAP, and B-CAP, respectively. The B-CAP CFR was 20%. ***Korea*** (three centers): 3151 CAP episodes were identified with 21.1% (20.4;21.7); 11.8% (11.2;12.3); 2.4% (2.1;2.7) due to S-CAP, C-CAP, and B-CAP, respectively. There were no deaths.

**Costs**: CAP-related hospitalization costs were highest for B-CAP episodes: 145.00 (Vietnam) to 1013.3 USD (Korea) per episode.

**Conclusion**: CAP hospitalization causes an important health and cost burden in all four countries studied (NMRR-12-50-10793).

## Introduction

Pneumonia is the leading cause of childhood deaths worldwide.^1^ In 2010 there were an estimated 120 million global episodes of pneumonia amongst children aged <5 years; in 2011 there were 1.3 million pneumonia deaths.^1-4^ Most childhood pneumonia occurs in low and middle income countries in Asia and Africa.^1^ In 2010 there were 47.4 million and 12.2 million episodes of childhood pneumonia in the World Health Organization (WHO) Southeast Asia and Western Pacific regions, respectively. Around 11% of the episodes in each region were hospitalized, and there were 443,800 and 61,900 deaths, respectively.^1,3^

The lack of a ‘gold standard’ in diagnosing pneumonia and establishing its etiology remains a major obstacle in determining the burden of pneumonia due to individual pathogens.^5^ Nevertheless, it is estimated that *Streptococcus pneumoniae (S. pneumoniae)* causes around one-third of childhood deaths due to pneumonia.^3,4,6^ Pneumococcal conjugate vaccines (PCVs) are immunogenic in young children and prevent a proportion of community-acquired pneumonia (CAP) caused by *S. pneumoniae*.^7-12^ Despite differences in settings, population, vaccine formulation, case definition and study design, estimates of PCV vaccine efficacy against radiologically-confirmed pneumonia with alveolar consolidation, in randomized controlled trials have been similar (range 22.0% to 35.1).^7-12^ Post-licensure studies in several countries have demonstrated significant reductions in hospital admissions for pneumonia among PCV-vaccinated children.^13-17^

The introduction of PCVs into routine childhood immunization schedules is one of several key measures identified by WHO to achieve a global reduction in childhood pneumonia.^18^ WHO recommends immunization using PCVs for all children, in conjunction with appropriate case management, breastfeeding and reducing risk factors, such as pollutant and tobacco smoke exposure.^19^ As epidemiological data are required to inform vaccination programs, efforts to characterize the burden of pneumonia in low income countries have been initiated.^20,21^

Data from the randomized controlled Clinical Otitis Media and Pneumonia Study (COMPAS), in which more than 10,000 children from middle-income countries in Latin America received the 10-valent PCV (PHiD-CV, *Synflorix*, GSK, Belgium) according to a 3-dose primary vaccination schedule, showed positive vaccine efficacy against CAP.^12^ Notably a vaccine efficacy of 22.0% (95% confidence intervals [CI] 7.7; 34.2) against likely bacterial CAP (B-CAP) in children aged <3 years was recorded. In order to extrapolate the results of the COMPAS study to Asia, data are required to characterize the burden of pneumonia using standardized case definitions. We therefore conducted a retrospective, hospital-based, cross-sectional study to estimate the burden of hospitalized CAP in children aged <5 years in four countries in the Southeast and Western Pacific Regions of Asia (Vietnam, Malaysia, Indonesia and Korea), using CAP case definitions aligned with those used in COMPAS. The extrapolation of PHiD-CV efficacy for the prevention of CAP from COMPAS study was conducted as a secondary endpoint for the hospitals located in Vietnam. These data will provide information for health care providers and policymakers considering national vaccination strategies.

## Results

### Vietnam

**Figure 1.**
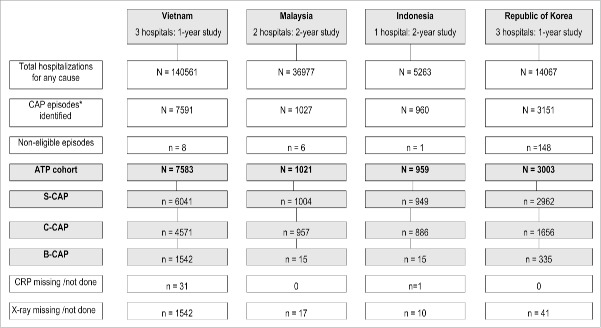
Total hospitalizations and CAP episodes in children <5 years of age, according to enrolled country. * Defined as any child aged <5 years hospitalized with any ICD-10 discharge code of interest. CAP, community acquired pneumonia; ATP, according to protocol; S-CAP, suspected CAP; C-CAP, confirmed CAP; B-CAP, bacterial CAP; CRP, C-reactive protein.

From January to December 2011, 6,041 hospitalized *suspected CAP* (S-CAP) episodes were identified in three study hospitals in Hue City, Hanoi and Ho Chi Minh City; 4,571 (75.7%) could be classified as *confirmed CAP* (C-CAP) and 1,542 (25.5%) as B-CAP (Fig. 1). An additional 1,542 CAP episodes could not be classified due to absent chest x-rays (CXR) results. Assuming that 25.5% of unclassifiable CAP episodes were indeed B-CAP, then the total estimated number of B-CAP cases was 1,935. S-CAP episodes represented 4.3% (6,041/140,561) (95% CI 4.2; 4.4), C-CAP episodes 3.3% (4,571/140,561) (95% CI 3.2; 3.4), and B-CAP episodes 1.4% (1,935/140,561) (95% CI 1.3; 1.4) of all-cause hospitalization in children aged <5 years.

In the hospital in Hue City, there were 282 S-CAP, 200 C-CAP and 10 B-CAP episodes during the study period, giving incidence rates of 11.7/1,000 person-years (95% CI 10.4; 13.1), 8.3/1,000 person-years (95% CI 7.2; 9.5), and 0.4/1,000 person-years (95% CI 0.2; 0.8), respectively. Assuming that 25.5% of episodes with absent CXR (n = 540) in Hue City were B-CAP episodes (n = 138), then the incidence of B-CAP was 6.1/1,000 person-years (95% CI 5.2; 7.2). There was a seasonal trend in CAP hospitalizations (any category) with two peaks; one in March and one in September.

Of 1,542 recorded B-CAP episodes identified in the three hospitals, 20 resulted in death (case fatality rate [CFR] 1.3%). In children with B-CAP, 749 (48.6%) recovered with sequelae (Table 1). The highest proportion of CAP admissions (any category) was in infants aged 7 weeks-12 months. 73.6% (4,443/6,043) of S-CAP episodes had C-reactive protein (CRP) testing performed (Sup Fig. 1). Among children with B-CAP, 974 (63.2%) of episodes were in males and a co-morbid condition was present in 262 (17.0%) of episodes (Sup Table 1). Only 0.1% of children with B-CAP or fewer were recorded as having received at least one dose of PCV, *Haemophilus influenzae* type b (Hib) or influenza vaccine (Sup Table 1).

**Table 1.** CAP outcome by country and CAP status (According-to-protocol cohort).

Country	Outcome	Suspected CAP	Confirmed CAP	Bacterial CAP	CRP missing/not done	Chest x-ray missing/not done
Vietnam		N = 6041	N = 4571	N = 1542	N = 3	N = 1542
	Fully recovered	2033 (33.7)	1618 (35.4)	619 (40.1)	0	402 (26.1)
	Recovered with sequelae	3490 (57.8)	2530 (55.4)	749 (48.6)	3 (100)	1094 (71.0)
	Discharged against medical advice	382 (6.3)	304 (6.7)	119 (7.7)	0	37 (2.4)
	Transferred to other hospital	95 (1.6)	83 (1.8)	30 (2.0)	0	6 (0.4)
	Death	27 (0.5)	25 (0.6)	20 (1.3)	0	1 (0.1)
	Unknown	14 (0.2)	11 (0.2)	5 (0.3)	0	2 (0.1)
Malaysia		N = 1004	N = 957	N = 15	N = 0	N = 17
	Fully recovered	892 (88.8)	859 (89.8)	12 (80.0)	—	13 (76.5)
	Recovered with sequelae	89 (8.9)	76 (7.9)	2 (13.3)	—	4 (23.5)
	Discharged against medical advice	8 (0.8)	7 (0.7)	0	—	0
	Transferred to other hospital	2 (0.2)	2 (0.2)	0	—	0
	Death	13 (1.3)	13 (1.4)	1 (6.7)	—	0
Indonesia		N = 949	N = 886	N = 15	N = 1	N = 10
	Fully recovered	749 (78.9)	694 (78.3)	8 (53.3)	1 (100)	8 (80.0)
	Recovered with sequelae	—	—	—	—	—
	Discharged against medical advice	146 (15.4)	139 (15.7)	4 (26.7)	0	1 (10.0)
	Transferred to other hospital	8 (0.8)	8 (0.9)	0	0	0
	Death	46 (4.9)	45 (5.1)	3 (20.0)	0	1 (10.0)
Korea		N = 2962	N = 1656	N = 335	N = 0	N = 41
	Fully recovered	2952 (99.7)	1647 (99.5)	332 (99.1)	—	41 (100)
	Recovered with sequelae	3 (0.1)	3 (0.2)	1 (0.3)	—	0
	Discharged against medical advice	2 (0.1)	1 (0.1)	1 (0.3)	—	0
	Transferred to other hospital	5 (0.2)	5 (0.3)	1 (0.3)	—	0
	Death	0	0	0	—	0

Suspected CAP = ICD-10 codes (J12-J86) with a referral for chest x-ray within first 3 days of hospitalization.

Confirmed CAP = Suspected CAP case who had a typical x-ray image of pneumonia (i.e. abnormal pulmonary infiltrates).

Bacterial CAP = Confirmed CAP case with CRP results ≥40 mg/l.

CRP missing/not done = Confirmed CAP case but with missing/not done CRP results, therefore impossible to classify further into B-CAP.

Chest x-ray missing/not done = Suspected CAP case in whom a chest X-ray was either not performed or was missing in the records, therefore impossible to classify further. N = number of episodes, % = n / Number of episodes with available results x 100.

CAP, community acquired pneumonia; CRP, C-reactive protein.

The median duration of hospitalization was 7 days (range: 1–66 days) across all categories of CAP. It was longest in infants ≤6 weeks of age and children 48–59 months of age (median 8 days, range 1–66 days) and shortest in 24–35 month olds (median 6 days, range 1–39 days).

The total median direct medical cost of B-CAP-related hospitalization was 145.00 USD, per episode (Table 2).

**Table 2.** Direct medical costs associated with CAP hospitalization in children in four countries in Asia.^*^

		S-CAP			C-CAP			B-CAP		
Country	Currency	N	Total median cost	Range	N	Total median cost	Range	N	Total median cost	Range
Vietnam	USD	595	90.60	0.30 to 3237.30	483	105.80	0.30 to 3237.30	170	145.00	2.9 to 2423.4
Malaysia	USD	349	109.40	43.50 to 1477.70	313	113.80	43.50 to 1477.70	3	375.80	332.40 to 537.10
Indonesia	USD	949	100.20	17.20 to 4933.00	886	99.10	17.20 to 4933.00	15	200.30	55.70 to 333.51
Korea	USD	2962	912.80	186.70 to 58,201.50	1656	967.50	291.00 to 23,614.10	335	1013.30	334.50 to 23,614.10
	Reimbursable Cost									
	USD		629.00	0.00 to 50,473.40		657.59	220.80 to 18,892.30		713.00	250.00 to 18,892.30
	Non-reimbursable cost									
	USD		267.80	0.00 to 7728.10		290.10	3.70 to 7640.10		288.20	19.40 to 5257.60

* Vietnam: 10% random sample. Malaysia: all subjects from one center and a 20% random sample from the second center.

Korea reimbursable cost = total cost of government subsidy for reimbursable items + total cost of patient out-of-pocket co-payment amount for reimbursable items.

CAP, community acquired pneumonia; S-CAP, suspected CAP; C-CAP, confirmed CAP; B-CAP, bacterial CAP; USD, United States dollars.

Applying the vaccine efficacy results from the COMPAS trial^12^ (22.0% against B-CAP) and assuming scenarios of 59% and 100% vaccine coverage, a vaccination program with PHiD-CV in Vietnam could potentially have prevented between 200.2 and 425.7 B-CAP hospitalizations and up to 5.5 B-CAP deaths (assuming all deaths were due to B-CAP) during the one-year study period in the three study centers (Table 3). Under the different scenarios PHiD-CV vaccination could have saved between 29,029 and 61,726 USD in direct medical costs in the study centers.

**Table 3.** Impact of the COMPAS efficacy results of 22.0% against B-CAP on B-CAP episodes and related deaths in study centers in Vietnam.

Identified B-CAP episodes			B-CAP hospitalizations prevented in study hospitals during the study period		
N episodes	N deaths	Vaccine coverage^*^	N episodes	N deaths	Direct medical costs saved^**^
1542.0	20.0	100%	339.2	4.4	49,184 USD
		59%	200.2	2.6	29,029 USD
1935.2^†^	25.2	100%	425.7	5.5	61,726 USD
		59%	251.2	3.3	36,424 USD

* 100% = best possible scenario, 59% = worst case scenario considering national DTP3 coverage of 2013.

** at 145 USD per B-CAP episode.

† assuming that 25.5% of unclassifiable S-CAP episodes were B-CAP episodes, with a case fatality rate of 1.3%.

CAP, community acquired pneumonia; B-CAP, bacterial CAP; COMPAS, Clinical Otitis Media and Pneumonia Study; N, number; USD, United States dollars.

Using incidence rates from Hue City, we estimated the overall annual hospitalized CAP burden in Vietnam to be 85,179 cases of hospitalized S-CAP; 60,436 cases of C-CAP and up to 44,597 cases of B-CAP. Under the different scenarios described above, a vaccination program with PHiD-CV in Vietnam could potentially have prevented between 5,789 and 9,811 B-CAP hospitalizations nationwide during the one-year study period.

### Malaysia

From December 2009 to December 2011, 1,004 hospitalized S-CAP episodes were identified in the two study hospitals in the cities of Serembam and of Kota Kinabalu; 957 (95.3%) of these could be classified as C-CAP and 15 (1.5%) as B-CAP episodes (Fig. 1). A further 17 episodes could not be further classified due to absent CXR results. Of note, only 3.8% (38/1,004) of S-CAP episodes had CRP testing performed (Table 4). The majority of children (97.5%) had one S-CAP episode over the study period. S-CAP episodes represented 2.7% (1,004/36,977) (95% CI 2.6; 2.9), C-CAP episodes 2.6% (957/36,977) (95% CI 2.4; 2.8) and B-CAP episodes 0.04% (15/36,977) (0.04; 0.1) of all-cause hospitalization in children aged <5 years. There were more CAP episodes during the first year of study than the second, with varying peaks observed around September.

**Table 4.** Summary of demographic information by CAP status for Malaysia (According-to-protocol cohort).

		Suspected CAP	Confirmed CAP	Bacterial CAP	Chest x-ray not done/missing	Total
Country/ variable	Category	n (%)	n (%)	n (%)	n (%)	n (%)
Malaysia		N = 1004	N = 957	N = 15	N = 17	N = 1021
CRP test done	Yes	38 (3.8)	36 (3.8)	15 (100)	0 (0.0)	38 (3.7)
Age group	≤6w	72 (7.2)	70 (7.3)	2 (13.3)	1 (5.9)	73 (7.1)
	7w-12m	469 (46.7)	457 (47.8)	6 (40.0)	5 (29.4)	474 (46.4)
	13m-23m	259 (25.8)	244 (25.5)	4 (26.7)	6 (35.3)	265 (26.0)
	24m-35m	109 (10.9)	101 (10.6)	3 (20.0)	2 (11.8)	111 (10.9)
	36m-47m	62 (6.2)	58 (6.1)	0 (0.0)	2 (11.8)	64 (6.3)
	48m-59m	33 (3.3)	27 (2.8)	0 (0.0)	1 (5.9)	34 (3.3)
Gender	Female	404 (40.2)	386 (40.3)	7 (46.7)	5 (29.4)	409 (40.1)
	Male	600 (59.8)	571 (59.7)	8 (53.3)	12 (70.6)	612 (59.9)
Weight for age	Above normal	25 (2.5)	23 (2.4)	0	0	25 (2.5)
	Normal	752 (74.9)	721 (75.3)	8 (53.3)	11 (64.7)	763 (74.7)
	Moderately underweight	128 (12.8)	120 (12.5)	1 (6.7)	5 (29.4)	133 (13.0)
	Severely underweight	99 (9.9)	93 (9.7)	6 (40.0)	1 (5.9)	100 (9.8)
	Missing/unknown	0	0	0	0	0
Vaccination history^a^	PCV	5 (0.5)	5 (0.5)	0 (0)	0 (0)	5 (0.5)
	Hib	802 (79.9)	760 (79.4)	9 (60.0)	15 (88.2)	817 (80.0)
	Influenza	8 (0.8)	8 (0.8)	1 (6.7)	0 (0)	8 (0.8)
Co-morbid conditions	Yes	86 (8.6)	86 (9.0)	6 (40.0)	2 (11.8)	88 (8.6)
	No	918 (91.4)	871 (91.0)	9 (60.0)	15 (88.2)	933 (91.4)

a Subjects who reported receiving at least one dose.

Suspected CAP = ICD-10 codes (J12-J86) with a referral for chest x-ray within first 3 days of hospitalization.

Confirmed CAP = Suspected CAP case who had a typical x-ray image of pneumonia (i.e. abnormal pulmonary infiltrates).

Bacterial CAP = Confirmed CAP case with CRP results ≥40 mg/l.

Chest x-ray not done/missing = Suspected CAP case but with chest x-ray not done/missing and unable to be classified further.

N = number of episodes, % = n / Number of episodes with available results x 100.

Above normal (overweight) (≥ +2 Z-score).

Normal weight (≥ −2 to < +2 Z-score).

Moderate underweight (≥ −3 to < −2 Z-score).

Severe underweight (< −3 Z-score).

CAP, community acquired pneumonia; CRP, C-reactive protein, Hib, *Haemophilus influenzae* type b vaccine; PCV, pneumococcal conjugate vaccines.

Of 15 B-CAP episodes, 12 children fully recovered and one died (Table 1). The highest proportion of CAP admissions (all CAP categories) was in children aged 7 weeks to 12 months (Sup Fig. 1). Among children with B-CAP, 53.3% were male (Table 4). A co-morbid condition was present in 9.0% of C-CAP episodes and in 40% of B-CAP episodes. Approximately 13.0% of children hospitalized for CAP were moderately underweight and 9.8% were severely underweight, including 40% with B-CAP. Only one child with B-CAP reported receiving at least one dose of influenza vaccine, none reported having received PCV vaccine, while 60.0% reported receiving at least one dose of Hib vaccine (Table 4).

The median duration of hospitalization was longest for B-CAP episodes (10 days, range 4–94) and shortest for S-CAP and C-CAP (median 4 days, range 1–94). Hospitalizations were longest in infants aged ≤6 weeks (median 8 days, range 2–43 days) and shortest in age groups above 24 months (median 3.0 days in each age stratum, range 1–31 days).

The total median direct medical cost of B-CAP-related hospitalization was 375.80 USD, per episode (Table 2).

### Indonesia

From January 2010 to November 2011, 949 hospitalized S-CAP episodes were identified in a hospital in the city of Bandung, 886 (93.4%) of which could be classified as C-CAP, and 15 (1.6%) as B-CAP (Fig. 1). Only 7.7% (73/949) of S-CAP episodes had CRP testing performed (Sup Table 2). A further 10 episodes lacked CXR results and could not be classified.

S-CAP episodes represented 18.0% (949/5,263) (95% CI 17.0, 19.1); C-CAP episodes 16.8% (886/5,263) (95% CI 15.8; 17.9) and B-CAP episodes 0.3% (15/5,263) (95% CI 0.2; 0.4) of all hospitalizations in children aged <5 years. The proportion of S-CAP hospitalizations (among all hospitalizations) was highest in children 7 weeks-12 months of age: 38.5% (95% CI 36.1; 40.9). There were 873 children (96.2%) who had one S-CAP episode. The number of CAP episodes peaked in March in each study year.

Among 15 B-CAP episodes, eight children (53.3%) fully recovered and three died (20%) (Table 1). There were more females (53.3% of episodes) than males admitted with B-CAP, and 26.7% of B-CAP episodes had a co-morbid condition at the time of admission. No children with B-CAP reported receiving any of the vaccines of interest (Sup Table 2). Approximately 20% of children hospitalized for B-CAP were moderately underweight and 13% were severely underweight (Sup Table 2).

The duration of hospitalization was longest for B-CAP episodes (median 10 days range 4–46) and shortest for S-CAP and C-CAP episodes (both median 6 days, range 1–71). Among S-CAP episodes, the duration of hospitalization was longest in infants aged ≤6 weeks (median 8 days range 2–25) and shortest in 48–59 month-olds (median 4.50, range 1–13). The majority of S-CAP episodes (63.1%) were in children between 7 weeks-12 months of age (Sup Fig. 1).

The total median direct medical cost of B-CAP-related hospitalization was 200.30 USD, per episode (Table 2).

### Republic of Korea

From January to December 2011, 2,962 hospitalized S-CAP episodes were identified in the three study hospitals, two located in Seoul and one in the city of Wonju, of which 1,656 (55.9%) were C-CAP and 335 (11.3%) were B-CAP episodes (Fig. 1). A further 41 S-CAP episodes could not be further classified due to absent CXR. Overall 99.0% (2,932/2,962) of S-CAP episodes had CRP testing performed. S-CAP episodes represented 21.1% (2,962/14,067) (95% CI 20.4; 21.7), C-CAP episodes 11.8% (1,656/14,067) (95% CI 11.2; 12.3) and B-CAP episodes 2.4% (335/14,067) (95% CI 2.1; 2.7) of all-cause hospitalizations in children aged <5 years. The proportion of S-CAP hospitalizations (among all hospitalizations) was highest in children 13–23 months of age (33.2%, 95% CI: 31.2; 35.3).

The majority of children (93.8%) had one CAP episode over the study period. The number of CAP episodes peaked in May and September to November. More than 99% of CAP episodes (all CAP categories) recovered and there were no deaths (Table 1).

The highest proportion of CAP admissions was in children aged 7 weeks-12 months and 13–23 months (Sup Fig. 1). Among B-CAP episodes, 51.9% were in male children, a co-morbid condition was present in 0.9% of episodes, and approximately 80% reported having received at least one dose of PCV or Hib vaccine (Sup Table 3).

The median duration of hospitalization was 4 days for S-CAP (range 1–141 days) and 5 days for C-CAP (range 1–38 days) and B-CAP (range 2–38 days), and was similar (median 4 or 5 days) in each age category.

The total median direct medical cost of B-CAP-related hospitalization was 1,013.30 USD per episode (Table 2). More than one-half of the total cost was reimbursable.

## Discussion

Hospital discharge databases were used to estimate the burden of hospitalized S-CAP, C-CAP and B-CAP among children <5 years of age in four countries in the Southeast and Western Pacific Regions of Asia. The four countries differ in terms of their healthcare systems (access to healthcare and health insurance, diagnostic and treatment capability), vaccination schedules and income status. Thus, while the CAP disease burden and associated direct medical costs were substantial in all four countries, observed differences in results are to be expected.

CAP hospitalizations as a proportion of all hospitalizations were highest in Korea (a high income country with a functioning health insurance system) and Indonesia (a middle income country with some areas of poverty), suggesting high incidence and/or low threshold for hospitalization in these countries. The proportion of CAP hospitalizations classifiable as B-CAP (more likely to be bacterial CAP) ranged from 1.5–25.5% across the countries, representing between <1% (Malaysia) and 1.4% (Vietnam) of all-cause hospitalizations in children aged <5 years during the study period. Elevated CRP was a diagnostic criteria for B-CAP in our study, yet CRP was tested in 64.0% of total CAP cases in Vietnam and in 7.8% or fewer cases in Indonesia and Malaysia (versus 99% in Korea), suggesting that B-CAP cases may have been significantly underestimated in these countries. In all countries, a higher percentage of hospitalized CAP episodes (all categories) were in children aged <24 months. However, the proportion of episodes in younger versus older children showed the least variability in Korea.

There were more deaths in children with B-CAP than with S-CAP or C-CAP, with the highest CFR rate observed in Indonesia; no deaths were recorded in Korea. A weight-for-age Z-score <-2 (moderate or severe underweight for age) has been linked to an increased risk of childhood pneumonia.^6^ In Indonesia and Malaysia, over 33% and 46% of hospitalized B-CAP episodes (albeit only 15 B-CAP cases in each country) were in children who were moderately or severely underweight, respectively, versus 2.4% (out of 335 B-CAP cases) in Korea. In addition, more B-CAP episodes occurred with co-morbid conditions in the low/middle-income countries in our study than in Korea (17% in Vietnam, 27% in Indonesia, 40% in Malaysia, versus 0.9% in Korea). Differing thresholds for hospitalization, lack of standardization and consistency of International Statistical Classification of Diseases and Related Health Problems (ICD) coding practices, differences in healthcare seeking behavior, and differences in insurance status of the patients are additional factors that could also contribute to the inter-country variability we observed in hospitalized CAP burden.

We were able to estimate the CAP incidence rate in children <5 years of age in Hue City, Vietnam for 2011. The incidence of B-CAP at this center is, in all probability, an underestimate due to the large number of unclassifiable CAP episodes (540/822, 65.7%): indeed B-CAP as a proportion of S-CAP was much lower in this center (10/282, 3.5%) than the other Vietnamese centers (1,542/6,041, 25.5%). Thus, the adjusted incidence rate of 6.1/1,000 person-years may be more likely to be nationally representative of B-CAP hospitalizations in children in Vietnam. However, our adjusted B-CAP estimate is higher than the results from a prospective population-based study in Nha Trang city, Vietnam in 2006, where the incidence of radiologically-confirmed hospitalized CAP in children aged <5 years was 3.3/1,000.^22^ Unlike our study, Yoshida et al^22^ undertook standardized CXR review, which may have led to more precise diagnoses and a lower level of case attribution. Also in our study for the cases for which a CXR could not be found despite a referral for a CXR, it may have been that these cases had a more mild disease presentation and that a CXR was not clinically required at the end. We were not able to compare the clinical features between those with missing CXRs and with CXRs available, so it is not clear whether the absence of CXR is a random event or whether this was a subgroup with a milder disease. Differences in catchment population estimates could also have influenced incidence rates in each study.

Due to the limited number of studies describing CAP burden in children in Asia, their slightly different definitions and patient recruitment from inpatient and/or outpatient settings, it is difficult to compare these findings with our observations. Nevertheless, the incidence of S-CAP in Hue city (11.7/1,000 person-years) is comparable to estimates for Southeast Asia (17.8/1,000 children per year) and the Western Pacific (17.3/1,000 children per year) in a recent literature review of severe (hospitalized) acute lower respiratory tract infection (LRTI) in children aged <5 years.^23^ Our estimate of 85,398 cases of hospitalized S-CAP among children <5 years in Vietnam is lower than 197,920 cases of severe LRTI (in all settings) in the same age group estimated by Rudan et al,^6^ which probably reflects that our study was limited to the hospital setting, and we used a different definition to identify cases.

Assuming PHiD-CV vaccine efficacy of 22.0% against B-CAP, PHiD-CV vaccination would have been expected to prevent up to 425.7 B-CAP hospitalizations, 5.5 deaths, and 61,726 USD over one year in the Vietnamese study hospitals. By extrapolating data from Hue City to the whole of Vietnam, we estimate that PHiD-CV vaccination could potentially have prevented between 5,789 and 9,811 B-CAP hospitalizations nationwide in 2011, or up to 1,422,644 USD.

There were few B-CAP episodes (<2% of all S-CAP) in the middle-income countries of Malaysia and Indonesia, probably reflecting the very low rates of CRP testing done in these centers. In the absence of denominator data (as the study hospitals' catchment areas in Indonesia and Malaysia were not well defined) we were unable to estimate CAP incidence rates, limiting comparisons to be made with other studies. However, some extrapolation and comparison of data between studies were possible. Aljunid et al, by using data from six hospitals in Malaysia, estimated that the incidence of pneumonia in children was 7.6/1,000 for all-cause pneumonia and 2.4/1,000 for pneumococcal pneumonia (2006-2007).^24^ We identified no studies of childhood CAP in Indonesia that considered a similar age range. However, the incidence of hospitalized pneumonia in children <2 years of age in rural Indonesia was 53/1,000 child years.^25^ Rudan et al estimated 450,611 episodes of severe lower respiratory tract infection in children <5 years of age in Indonesia in 2010, of which around 18.3% were due to *S. pneumoniae*.^6^ Thus it seems very likely that the B-CAP burden in Malaysia and Indonesia in our study have been substantially underestimated.

Data from other middle-income countries also indirectly suggest that our study may have underestimated the B-CAP burden in Malaysia and Indonesia: In a large population-based study (2005-2010) the incidence of hospitalized acute LRTI in children <5 years of age in Thailand was estimated to be 57.7/1,000 person-years, with a CFR of 0.3%.^26^ Among children <5 years of age in Uruguay, the incidence of consolidated pneumonia was 11.75/1,000 per year (2001-2004).^27^

PCVs were not included in the national immunization programs (NIP) of any of countries at the time of the study, but were available in the private market. Approximately 78% of children in Korea had received at least one PCV dose, which is in line with published data (>74% of the 2010 Korean birth cohort received the PCV primary series).^28^ By contrast, the number of children who had received a PCV in the other study countries was negligible. Despite high PCV coverage in Korea, we observed the highest proportion of B-CAP hospitalizations among all-cause hospitalizations in Korea, which may also partly reflect the CRP testing of almost all (99%) CAP cases. In a study that estimated childhood pneumonia cases in 192 countries in 2011 (including Korea), Rudan et al. also noted that a higher proportion of pneumonia cases was hospitalized in high-income countries as compared to low- and middle-income countries.^6^ The authors postulated that this could be due to a lower clinical threshold for hospitalizing children in high-income countries, or, by a proportionally greater reduction in clinically mild pneumonia than severe pneumonia in lower income countries, due to the effects of improved social conditions. This observation is also consistent with WHO recommendations to treat non-severe pneumonia episodes in challenging settings (low- and middle-income countries) in the community, with hospitalization reserved for severe cases.^29^

Very small proportions (<1%) of children in Vietnam and Indonesia had received Hib vaccination, compared with over 75% in Malaysia and Korea. Hib-containing vaccines were included in the Malaysian NIP in 2002, Vietnam in June 2010, and not until after our study in Korea (2013) and Indonesia (2014). The unexpectedly low Hib vaccination rates reported in Vietnam may reflect incomplete recording of Hib vaccination in hospital medical records.

The disease CAP was associated with a significant cost burden in all countries. The highest median direct medical costs in all study sites were associated with B-CAP hospitalizations. Due to the potential differences in the case-definition, sample size and geographic locations, we were not able to make direct comparisons of the cost data from our study with those reported in the literature from different countries. However, we have noted that our results were comparable with, or within a reasonable range of the data reported in published studies from Vietnam, Malaysia and Korea.^24,30,31^ Apart from the economic burden driven by direct medical costs, hospitalized pneumonia cases in children have also been reported to have a substantial impact on the family and society due to direct non-medical costs and indirect costs. A recent study in Vietnam reported that direct non-medical and indirect costs for a hospitalized pneumonia case for children aged <5 years were approximately 62 USD (2012 value).^31^ Reported direct costs associated with hospitalized pneumonia of 180 USD (2012 costs), account for almost 30% of the total costs of hospitalized cases of pneumonia.^30,31^ As expected, similar proportions (non-medical and indirect costs versus direct costs) were observed in developed countries in the region, probably representing work loss and transportation costs involved when taking care of a child hospitalized for pneumonia. In Taiwan, the proportion was about 37% (222 USD, 2009 value).^32^ Our study did not consider direct non-medical costs or indirect costs to households. Our cost estimate of 1,210.26 MR (376 USD) for the pneumonia burden in Malaysia is lower than the cost estimate of 2,592 MR (754 USD, 2007 data) for hospitalized all-cause pneumonia in children reported in another study.^24^ This is because Aljunid et al. included capital costs, costs of labour, supplies and utilities, which we did not include in our study. In Korea, our cost estimate for pneumonia burden (1,069,316 KW/1,043 USD) is higher than the estimate of 690,000 KW (726 USD) for hospitalised pneumonia (2006 prices) reported by Sohn et al.^30^

The strengths of our study include the large number of medical records reviewed for CAP episodes; the use of a single harmonized protocol between the four countries which allowed application of the same CAP definitions, data collection methods, and statistical analysis; the geographical distribution of sites in Vietnam, Korea and Malaysia offering good representation of the countries studied; the availability of one site where incidence could be estimated; and the collection of cost data providing additional insight into the CAP burden in the four countries. Nevertheless, in view of differences in health care systems and potential differences in disease management, health care seeking behaviors, and ICD 10th Revision (ICD-10) coding procedures between individual countries, comparisons across countries should be avoided and our study may have limited applicability to other regions. Even if the 4 countries may differ in terms of healthcare and delivery systems and healthcare seeking behaviors, they are part of Asia and the overall results of this study suggest that independently of the health care system considered, there is a significant number of hospitalizations attributable to community acquired pneumonia (varying between 2.7% in Malaysia to 21.1% in Korea) and there is an appreciable burden of healthcare costs associated with these episodes of hospitalizations (ranging from 145 USD per episode of hospitalization in Vietnam to 1013 USD in Korea).

The single study center in Indonesia means any extrapolation to the national level should be undertaken cautiously, while the predominately urban locations studied potentially limit the generalizability to rural populations. Our study focused on hospitalized cases, but in low/middle- and middle-income countries many pneumonia cases may not access hospital care, and more than 80% of deaths may occur outside hospital.^25,33^ Thus our study is unlikely to reflect the full burden of childhood pneumonia in the studied countries.

There was a lack of CRP testing, which precluded a diagnosis of B-CAP in most children in Indonesia and Malaysia, and another potential limitation of the study was that the study duration in Vietnam and in the Republic of Korea could only last for one year and this because of logistical difficulties. We applied a retrospective design relying on individual physicians' diagnosis of pneumonia (and ICD-10 coding) in each of the centers. Therefore no uniform diagnostic criteria for pneumonia were applied. We were unable to assess any impact of coding misclassification in any of the centers. We evaluated CXR results using capture words (e.g., consolidation) and did not conduct an expert review, which may have over- or under-estimated the proportion of B-CAP episodes identified. We decided to extrapolate the PHiD-CV efficacy for prevention of CAP from COMPAS to Vietnam only, as the CRP laboratory test was not widely used in Malaysia and Indonesia to classify the cases. No data on the circulating *S. pneumoniae* serotypes were published from the population covered by the study sites in Korea at the time of the protocol development of this study, as well as also the catchment population of the hospital sites in Korea could not be defined. Finally, the analysis of cost data was limited to a subset of validated episodes in Vietnam and Malaysia. Only direct medical costs were assessed in the study, which only provides a fraction of the total costs associated with pediatric pneumonia hospitalizations.

This is one of few studies to assess the burden of hospitalized CAP in children living in the Western Pacific and Southeast Asia region. While there are apparently marked differences between countries in terms of the CAP burden and the characteristics of children who are admitted to hospital with CAP, the study indicates an important CAP burden in all of the countries studied. The few published studies estimating the pneumonia burden in the region differ in terms of setting, age group studied, and CAP case definition used, making comparisons between studies challenging. There is also a need for surveillance studies to be conducted in Malaysia, Vietnam, and Indonesia that are based on active, prospective enrolment of children suffering from CAP in hospital and community sites. Ideally these studies should be conducted in defined populations to allow accurate estimates of incidence to be made. Alternatively, studies to validate the performance of the coded discharge diagnoses in these countries, in order to accurately and completely identify all CAP cases in children, would provide valuable information.

Vaccination is identified as one of the key interventions in pneumonia prevention, that could reduce the observed health inequalities between countries, and reduce morbidity and mortality due to CAP.^34,35^ Therefore decisions have to be made about the value and affordability of investing limited resources into research to investigate the CAP burden before investing in vaccination, or immediate investment in vaccination strategies to address this public health issue. In the second scenario, monitoring the CAP disease burden in the post-vaccination period could evaluate the real-life performance of vaccination, thus ensuring that optimal prevention methods against CAP in children are rapidly implemented.

## Methods

### Study design

The primary study objective was to estimate the proportion of all hospitalizations in children aged <5 years that could be attributed to CAP (Malaysian registry number NMRR-12-50-10793). The clinical outcome of CAP episodes, duration of CAP hospitalizations, and the cost burden were also assessed. The incidence of hospitalization due to CAP was determined for Hue City Vietnam, which had a defined hospital catchment area. The study period lasted 1 year (January-December 2011) in Vietnam and Korea, but lasted 2 years (January 2010-December 2011) in Indonesia and Malaysia. For Vietnam, where incidence rates could be calculated, the number of hospitalizations, deaths, and costs associated with B-CAP, and potentially preventable by vaccination with PHiD-CV were estimated by applying the COMPAS trial results^15^ to the Vietnamese data. A secondary endpoint of our study included the extrapolation of PHiD-CV efficacy for prevention of CAP from COMPAS study to Vietnam.

The study was conducted according to the Declaration of Helsinki and Good Clinical Practice guidelines. The study protocol and associated documents were reviewed and approved by institutional review boards in each participating country. All data were anonymized and informed consent was not required.

### Study setting and socioeconomic indicators

The study was conducted in nine hospitals in four countries: three referral hospitals in Vietnam (Hue Central Hospital in Hue City, located in the middle of the country, Children Hospital No.1 in Ho Chi Minh City, located in the south of the country, and Hanoi's National Hospital of Pediatrics, located in the north); two referral hospitals in Malaysia (Tuanku Ja'afar Hospital in Seremban, Negeri Sembilan state on the western coast, and Likas Hospital in Kota Kinabalu, Sabah state in East Malaysia); one tertiary referral hospital in Indonesia (Hasan Sadikin General Hospital located in Bandung in West Java); and three referral hospitals in Korea (Wonju Severance Christian Hospital in Gangwon-do province in the center of the country; and Severance Children's Hospital and Gangnam Severance Hospital, both in Seoul).

The coverage of routine pediatric vaccines varies in Vietnam (95% in 2011 for the third infant dose of diphtheria-tetanus-pertussis vaccine [DTP3]; but decreased to 59% for DTP3 in 2013), but is consistently high in Indonesia, Malaysia and Korea (range 86% to >99% for DTP3) (2013 data).^20^ At the time of the study, none of the studied countries included PCVs or influenza vaccination for children in their NIP. However, in all countries except Vietnam, a PCV was registered and used in the private sector (paid out-of-pocket). Hib vaccination was recommended in Vietnam (from June 2010), Malaysia and Korea.^36^

The four studied countries are economically diverse. The World Bank classifies Vietnam as a low/middle-income country, Malaysia as an upper middle-income country, Indonesia as a lower middle-income country, and Korea as a high-income country.^37^ Infant mortality rates are lower in each country than the global rate of 34.9 per 1,000 live births, but ranged between 3.3 per 1,000 live births in Korea to 25.8 per 1,000 live births in Indonesia.^38^

### Data sources and collection

Medical records of participating hospitals were reviewed to identify children aged <5 years with diagnosed CAP, using the ICD-10 codes J12-J22, J85-J86 (all cause pneumonia, pneumonia due to *S. pneumoniae*, pneumonia due to Hib, unspecified bacterial pneumonia and pneumonia-organism unspecified; presented in supplemental digital content). Medical records were selected on the basis of first and any listed ICD-10 codes relating to pneumonia. Information on demographics, duration of hospitalization, outcome of hospitalization, medical history (previous upper respiratory tract infection, hay fever, and asthma), nutritional status, vaccination history (Hib, influenza and/or PCV vaccination) and the presence of any comorbid conditions (e.g., cardio-vascular, neurological, malignancies, congenital defects…) were extracted from medical records. CRP levels and results from CXR were also retrieved from medical records in order to categorize CAP in the analysis.

Hospital administrative databases were used to retrospectively collect unsubsidized total direct medical costs of CAP-related hospitalizations in Vietnam, Indonesia and Korea. In Malaysia, as unsubsidized total costs were not available from the hospital database, medical resource use (including ward stay, radiology testing, white blood cell count, blood culture and antibiotics) and itemized costs of the corresponding resource were retrieved from the hospital database and Ministry of Health website respectively, to compute the total costs.

For incidence estimation, all enrolled subjects with ‘Hue’ as their city of residence were included for the numerator. Data on the total number of children aged <5 years living in Hue City in 2011 were obtained from the official Hue City website (http://www.huecity.gov.vn) and used as the denominator. The total population of children <5 years of age in Vietnam was estimated from United Nations statistics for 2010.^39^

### Study population and CAP case definition

The study population included data from all children aged <5 years who were hospitalized for pneumonia in the participating hospitals. Children hospitalized more than once during the study period were considered as new cases if they had had a 30-day symptom-free interval between the dates of discharge and new admission.

The identified ICD-10 codes were matched to the CAP case definitions in COMPAS.^12^ S-CAP was defined as having any of the discharge ICD-10 codes J12-J86 with a referral for CXR within the first 3 days of hospitalization; C-CAP was defined as an S-CAP case with abnormal pulmonary infiltrates on CXR; and B-CAP defined as a radiologically-confirmed CAP case with a CXR showing either alveolar consolidation/pleural effusion, or non-consolidated pneumonia accompanied by CRP level ≥40 mg/L. The CAP status definitions were considered mutually inclusive. Cases of nosocomially-acquired pneumonia (defined as the onset of signs and symptoms ≥3 days of hospitalization) were excluded from the analysis.

### Statistical analysis

The according-to-protocol cohort included all children who met the inclusion/exclusion criteria and for whom data were available. Analyses were performed per country.

The proportion of hospitalizations (and exact 95% CI) due to CAP among all hospitalizations in children aged <5 years was estimated using the total number of hospitalizations during the study period as the denominator. The proportion of S-CAP, C-CAP and B-CAP cases was estimated by age group (≤6 weeks, 7 weeks-12 months, 13–23 months, 24–35 months, 36–47 months, 48–59 months), gender, month and year of hospitalization. Weight-for-age z-scores were computed using WHO criteria^40^ to evaluate nutritional status; *Moderately underweight* for age was defined as a z-score ≥-3 and <-2. *Severely underweight* for age was defined as a z-score <-3.

The incidence (and exact 95% CI) of CAP episodes in hospitalized children in Hue City, Vietnam was calculated by CAP status. The number of hospitalizations and deaths from B-CAP potentially prevented, and costs potentially saved by vaccination with PHiD-CV in Vietnam were estimated by applying the vaccine efficacy results from COMPAS^12^ to the observed data. In the first scenario, vaccine coverage was assumed to be 100% and in the second, was assumed to be the same as for DTP3 in Vietnam (59%).

Median total direct medical costs of CAP-related hospitalizations were calculated per country and adjusted to 2011 values using the Consumer Price Index of the country, wherever applicable. In Vietnam, cost data were analyzed for a 10% random sample taken across all three centers (n = 595). In Malaysia, cost data were analyzed for 20% of randomly selected CAP episodes from one center and for all episodes from the other center (n = 349). In Korea, unsubsidized total direct medical costs, associated costs for reimbursable items (government subsidy and patient out-of-pocket amounts) and non-reimbursable items were collected from hospital administrative databases from all three centers. Costs were presented in USD (converted using http://www.oanda.com/currency/converter/ in December 2013).

We assumed that 25% of all pediatric hospital admissions were due to lower respiratory tract infections (LRTI) as a proxy for pneumonia or CAP.^41^ In the absence of precise data on CAP hospitalizations as a proportion of all-cause hospitalizations in the region, we calculated that the exact 95% CI around the proportion of CAP hospitalizations among children <5 years of age would be (24.6%; 25.4%) in an arbitrarily selected population of 50,000 all-cause hospitalizations.

The statistical analysis used *SAS Version 9.2* with Drug and Development web portal version 3.5.

## Supplementary Material

Supplemental_Material.zip

## Trademark section

Synflorix is a trade mark owned by or licensed to the GSK group of companies.
