# Supplementary material for: Burden of hospitalized childhood community-acquired pneumonia: A retrospective cross-sectional study in Vietnam, Malaysia, Indonesia and the Republic of Korea
Source: Hum Vaccin Immunother. 2017 Nov 10;14(1):95–105. doi: 10.1080/21645515.2017.1375073 (PMC5791577; doi:10.1080/21645515.2017.1375073)
Supplement: Supplemental_Material.zip [file khvi-14-01-1375073-s001.zip › Supplemental Material.docx]

**Supplemental digital content data**

**International Statistical Classification of Diseases and Related Health Problems 10th Revision (ICD-10) codes used for case identification**

The following ICD codes were used in the study:

- J12- Viral pneumonia, not elsewhere classified.
- J13- Pneumonia due to *Streptococcus* *pneumoniae.*
- J14- Pneumonia due to *Haemophilus influenzae*.
- J15- Bacterial pneumonia, not elsewhere classified.
- J16- Pneumonia due to other infectious organisms, not elsewhere classified.
- J17- Pneumonia in diseases classified elsewhere.
- J18- Pneumonia, unspecified organism.
- J20- Acute bronchitis.
- J21- Acute bronchiolitis.
- J22- Unspecified acute lower respiratory infection.
- J85- Abscess of lung and mediastinum.
- J86- Pyothorax.

**Supplemental files**

**Sup Figure 1:** Age distribution of hospitalized CAP by country (According-to-protocol cohort).

Footnote: CAP, community acquired pneumonia

**Sup Table 1:** Summary of demographic information by CAP status in Vietnam (According-to-protocol cohort)

| **Country/**  **variable** | **Category** | **Suspected CAP** | **Confirmed CAP** | **Bacterial CAP** | **Chest**  **x-ray missing/not done** | **Total** |
| --- | --- | --- | --- | --- | --- | --- |
|  |  | **n (%)** | **n (%)** | **n (%)** | **n (%)** | **n (%)** |
| **Vietnam** |  | **N = 6041** | **N = 4571** | **N = 1542** | **N = 1542** | **N = 7583** |
| CRP test done | Yes | 4443 (73.6) | 3588 (78.5) | 1542 (100) | 406 (26.3) | 4849 (64.0) |
| Age group | ≤6w | 1205 (19.9) | 856 (18.7) | 306 (19.8) | 135 (8.8) | 1340 (17.7) |
|  | 7w-12m | 2642 (43.7) | 2015 (44.1) | 731 (47.4) | 749 (48.6) | 3391 (44.7) |
|  | 13m-23m | 1181 (19.5) | 903 (19.8) | 282 (18,3) | 303 (19.6) | 1484 (19.6) |
|  | 24m-35m | 676 (11.2) | 543 (11.9) | 147 (9.5) | 221 (14,3) | 897 (11.8) |
|  | 36m-47m | 236 (3.9) | 177 (3.9) | 51 (3.3) | 105 (6.8) | 341 (4.5) |
|  | 48m-59m | 101 (1.7) | 77 (1.7) | 25 (1.6) | 29 (1.9) | 130 (1.7) |
| Gender | Female | 2273 (37.6) | 1719 (37.6) | 568 (36.8) | 579 (37.5) | 2852 (37.6) |
|  | Male | 3768 (62.4) | 2852 (62.4) | 974 (63.2) | 963 (62.5) | 4731 (62.4) |
| Weight for age | Above normal | 280 (4.7) | 199 (4.4) | 71 (4.6) | 61 (4.0) | 341 (4.5) |
|  | Normal | 5048 (84.3) | 3847 (84.9) | 1302 (84.8) | 1269 (83.4) | 6317 (84.1) |
|  | Moderately underweight | 411 (6.9) | 299 (6.6) | 104 (6.8) | 128 (8.4) | 539 (7.2) |
|  | Severely underweight | 252 (4.2) | 189 (4.2) | 58 (3.8) | 64 (4.2) | 316 (4.2) |
|  | Missing/unknown | 50 | 37 | 7 | 20 | 70 |
| Vaccination history^a^ | PCV | 3 (0.1) | 2 (0.04) | 2 (0.1) | 2 (0.13) | 5 (0.07) |
|  | Hib | 6 (0.1) | 4 (0.09) | 2 (0.1) | 1 (0.06) | 7 (0.09) |
|  | Influenza | 3 (0.05) | 1 (0.02) | 0 (0) | 3 (0.2) | 6 (0.08) |
| Co-morbid conditions | Yes | 956 (15.8) | 720 (15.8) | 262 (17.0) | 175 (11.4) | 1131 (14.9) |
|  | No | 5085 (84.2) | 3851 (84.3) | 1280 (83.0) | 1367 (88.7) | 6452 (85.1) |

^a^ Subjects who reported receiving at least one dose

Suspected CAP = ICD-10 codes (J12-J86) with a referral for chest x-ray within first 3 days of hospitalization

Confirmed CAP = Suspected CAP case who had a typical x-ray image of pneumonia (i.e. abnormal pulmonary infiltrates)

Bacterial CAP = Confirmed CAP case with CRP results ≥40 mg/l

CRP missing = Confirmed CAP case but with missing CRP results, therefore impossible to classify further into B-CAP.

Chest x-ray missing/not done= Suspected CAP case but with chest x-ray results missing/not done, therefore impossible to classify further into C- or B-CAP

N = number of episodes, % = n / Number of episodes with available results x 100

Above normal (overweight) (≥ +2 Z-score)

Normal weight (≥ -2 to < +2 Z-score)

Moderate underweight (≥ -3 to < -2 Z-score)

Severe underweight (< -3 Z-score)

CAP, community acquired pneumonia; CRP, C-reactive protein, Hib, *Haemophilus influenzae* type b vaccine; PCV, pneumococcal conjugate vaccines

**Sup Table 2:** Summary of demographic information by CAP status for Indonesia (According-to-protocol cohort)

| **Country/**  **variable** | **Category** | **Suspected CAP** | **Confirmed CAP** | **Bacterial CAP** | **Chest**  **x-ray missing/not done** | **Total** |
| --- | --- | --- | --- | --- | --- | --- |
|  |  | **n (%)** | **n (%)** | **n (%)** | **n (%)** | **n (%)** |
| **Indonesia** |  | **N = 949** | **N = 886** | **N = 15** | **N = 10** | **N = 959** |
| CRP test done | Yes | 73 (7.7) | 68 (7.7) | 15 (100) | 2 (20.0) | 75 (7.8) |
| Age group | ≤6w | 76 (8.0) | 71 (8.0) | 2 (13.3) | 2 (20.0) | 78 (8.1) |
|  | 7w-12m | 599 (63.1) | 567 (64.0) | 10 (66.7) | 4 (40.0) | 603 (62.9) |
|  | 13m-23m | 163 (17.2) | 149 (16.8) | 2 (13.3) | 2 (20.0) | 165 (17.2) |
|  | 24m-35m | 57 (6.0) | 51 (5.8) | 1 (6.7) | 0 (0.0) | 57 (5.9) |
|  | 36m-47m | 29 (3.1) | 24 (2.7) | 0 (0) | 1 (10.0) | 30 (3.1) |
|  | 48m-59m | 25 (2.6) | 24 (2.7) | 0 (0) | 1 (10.0) | 26 (2.7) |
| Gender | Female | 417 (43.9) | 390 (44.0) | 8 (53.3) | 6 (60.0) | 423 (44.1) |
|  | Male | 532 (56.1) | 496 (56.0) | 7 (46.7) | 4 (40.0) | 536 (55.9) |
| Weight for age | Above normal | 22 (2.3) | 21 (2.4) | 0 (0) | 1 (10.0) | 23 (2.4) |
|  | Normal | 561 (59.7) | 520 (59.2) | 10 (66.7) | 5 (50.0) | 566 (59.6) |
|  | Moderately underweight | 160 (17.0) | 154 (17.5) | 3 (20.0) | 2 (20.0) | 162 (17.1) |
|  | Severely underweight | 197 (21.0) | 183 (20.8) | 2 (13.3) | 2 (20.0) | 199 (21.0) |
|  | Missing/unknown | 9 | 8 | 0 | 0 | 9 |
| Vaccination history^a^ | PCV | 3 (0.3) | 2 (0.2) | 0 (0) | 0 (0) | 3 (0.3) |
|  | Hib | 1 (0.1) | 0 (0) | 0 (0) | 0 (0) | 1 (0.1) |
|  | Influenza | 1 (0.1) | 0 (0) | 0 (0) | 0 (0) | 1 (0.1) |
| Co-morbid conditions | Yes | 209 (22.0) | 186 (21.0) | 4 (26.7) | 2 (20.0) | 211 (22.0) |
|  | No | 740 (78.0) | 700 (79.0) | 11 (73.3) | 8 (80.0) | 748 (78.0) |

^a^ Subjects who reported receiving at least one dose

Suspected CAP = ICD-10 codes (J12-J86) with a referral for chest x- ray within first 3 days of hospitalization

Confirmed CAP = Suspected CAP case who had a typical x- ray image of pneumonia (i.e. abnormal pulmonary infiltrates)

Bacterial CAP = Confirmed CAP case with CRP results ≥40 mg/l

Chest x-ray not done/missing = Suspected CAP case nu twith chest x-ray lacking and unable to be classified further

N = number of episodes, % = n / Number of episodes with available results x 100

Above normal (overweight) (≥ +2 Z-score)

Normal weight (≥ -2 to < +2 Z-score)

Moderate underweight (≥ -3 to < -2 Z-score)

Severe underweight (< -3 Z-score)

CAP, community acquired pneumonia; CRP, C-reactive protein, Hib, *Haemophilus influenzae* type b vaccine; PCV, pneumococcal conjugate vaccines

**Sup Table 3:** Summary of demographic information by CAP status for the Republic of Korea (According-to-protocol cohort)

| **Country/**  **variable** | **Category** | **Suspected CAP** | **Confirmed CAP** | **Bacterial CAP** | **Chest**  **x-ray not done/missing** | **Total** |
| --- | --- | --- | --- | --- | --- | --- |
|  |  | **n (%)** | **n (%)** | **n (%)** | **n (%)** | **n (%)** |
| **Korea** |  | **N = 2962** | **N = 1656** | **N = 335** | **N = 41** | **N = 3003** |
| CRP test done | Yes | 2932 (99.0) | 1646 (99.4) | 335 (100) | 41 (100) | 2973 (99.0) |
| Age group | ≤6w | 109 (3.7) | 51 (3.1) | 4 (1.2) | 2 (4.9) | 111 (3.7) |
|  | 7w-12m | 743 (25.1) | 316 (19.1) | 46 (13.7) | 14 (34.2) | 757 (25.2) |
|  | 13m-23m | 713 (24.1) | 379 (22.9) | 71 (21.2) | 8 (19.5) | 721 (24.0) |
|  | 24m-35m | 565 (19.1) | 338 (20.4) | 66 (19.7) | 6 (14.6) | 571 (19.0) |
|  | 36m-47m | 472 (15.9) | 312 (18.8) | 76 (22.7) | 5 (12.2) | 477 (15.9) |
|  | 48m-59m | 360 (12.2) | 260 (15.7) | 72 (21.5) | 6 (14.6) | 366 (12.2) |
| Gender | Female | 1313 (44.3) | 764 (46.1) | 161 (48.1) | 20 (48.8) | 1333 (44.4) |
|  | Male | 1649 (55.7) | 892 (53.9) | 174 (51.9) | 21 (51.2) | 1670 (55.6) |
| Weight for age | Above normal | 176 (6.0) | 78 (4.7) | 13 (3.9) | 0 (0) | 176 (5.9) |
|  | Normal | 2669 (90.5) | 1524 (92.3) | 313 (93.7) | 41 (100) | 2710 (90.7) |
|  | Moderately underweight | 54 (1.8) | 25 (1.5) | 4 (1.2) | 0 (0) | 54 (1.8) |
|  | Severely underweight | 49 (1.7) | 24 (1.5) | 4 (1.2) | 0 (0) | 49 (1.6) |
|  | Missing/unknown | 14 | 5 | 1 | 0 | 14 |
| Vaccination history^a^ | PCV | 2323 (78.4) | 1338 (80.8) | 272 (81.2) | 31 (75.6) | 2354 (78.4) |
|  | Hib | 2332 (78.7) | 1337 (80.7) | 269 (80.3) | 31 (75.6) | 2363 (78.7) |
|  | Influenza | 1060 (35.8) | 594 (35.9) | 117 (34.9) | 18 (43.9) | 1078 (35.9) |
| Co-morbid conditions | Yes | 28 (1.0) | 15 (0.9) | 3 (0.9) | 0 (0) | 28 (0.9) |
|  | No | 2934 (99.1) | 1641 (99.1) | 332 (99.1) | 41 (100) | 2975 (99.1) |

^a^ Subjects who reported receiving at least one dose

Suspected CAP = ICD-10 codes (J12-J86) with a referral for chest x-ray within first 3 days of hospitalization

Confirmed CAP = Suspected CAP case who had a typical x-ray image of pneumonia (i.e. abnormal pulmonary infiltrates)

Bacterial CAP = Confirmed CAP case with CRP results ≥40 mg/l

Chest x-ray not done/missing = Suspected CAP case with chest x-ray not done/missing and unable to be classified further

N = number of episodes, % = n / Number of episodes with available results x 100

Above normal (overweight) (≥ +2 Z-score)

Normal weight (≥ -2 to < +2 Z-score)

Moderate underweight (≥ -3 to < -2 Z-score)

Severe underweight (< -3 Z-score)

CAP, community acquired pneumonia; CRP, C-reactive protein, Hib, *Haemophilus influenzae* type b vaccine; PCV, pneumococcal conjugate vaccines
