# Supplementary figures and images for: Burden of hospitalized childhood community-acquired pneumonia: A retrospective cross-sectional study in Vietnam, Malaysia, Indonesia and the Republic of Korea
Source: Hum Vaccin Immunother. 2017 Nov 10;14(1):95–105. doi: 10.1080/21645515.2017.1375073 (PMC5791577; doi:10.1080/21645515.2017.1375073)

Sup Figure 1

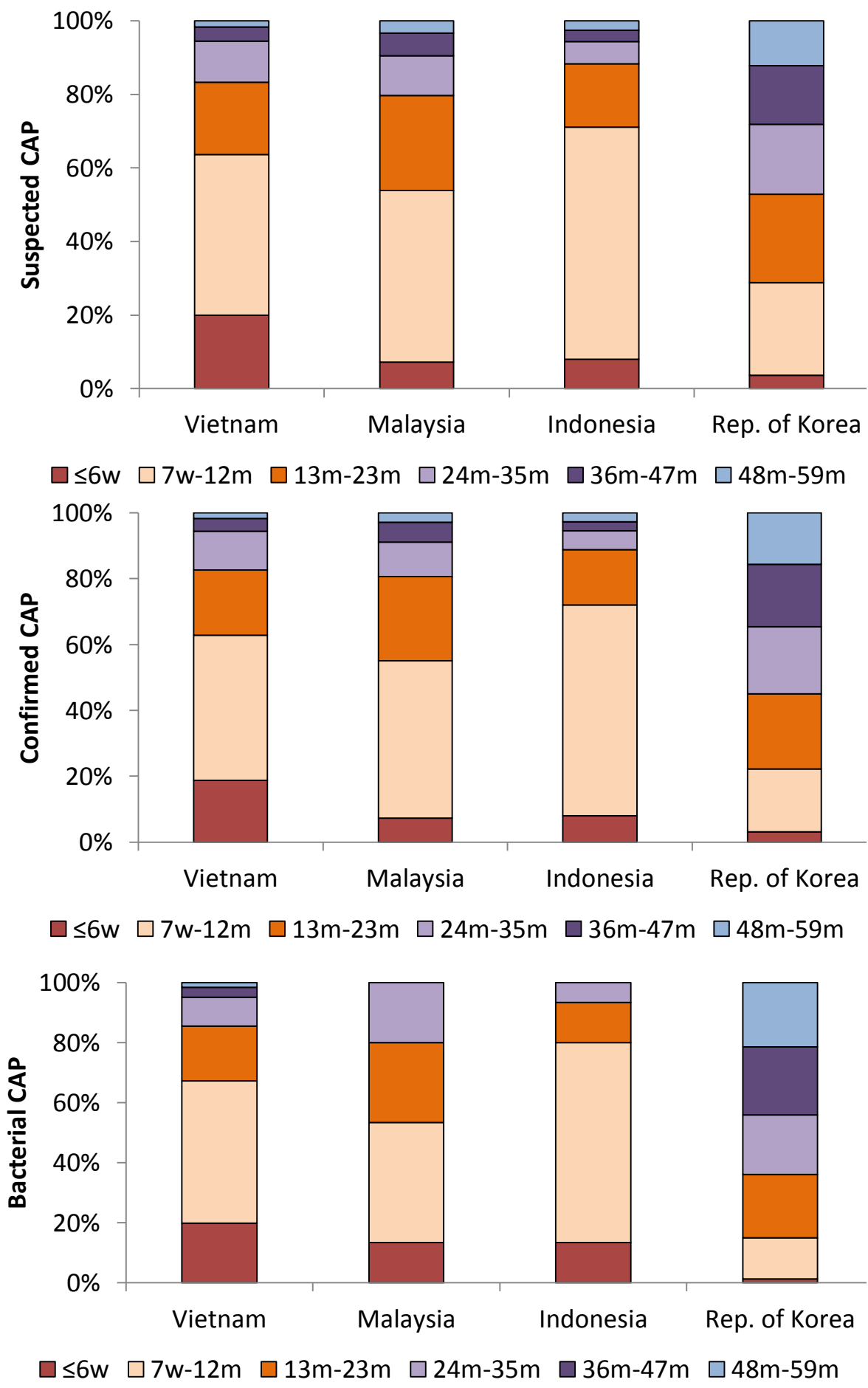

Supplement: Supplemental_Material.zip [file khvi-14-01-1375073-s001.zip › 2017HV0070R-s02.pdf]
